# Insular and limbic abnormal functional connectivity in early-stage Parkinson’s disease patients with autonomic dysfunction

**DOI:** 10.1093/cercor/bhae270

**Published:** 2024-07-05

**Authors:** Matteo Conti, Elena Garasto, Roberta Bovenzi, Valerio Ferrari, Nicola B Mercuri, Francesca Di Giuliano, Rocco Cerroni, Mariangela Pierantozzi, Tommaso Schirinzi, Alessandro Stefani, Camilla Rocchi

**Affiliations:** Neurology Unit, Department of Systems Medicine, University of Rome “Tor Vergata”, Via Montpellier 1, 00133 Rome, Italy; Neurology Unit, Department of Systems Medicine, University of Rome “Tor Vergata”, Via Montpellier 1, 00133 Rome, Italy; Neurology Unit, Department of Systems Medicine, University of Rome “Tor Vergata”, Via Montpellier 1, 00133 Rome, Italy; Neurology Unit, Department of Systems Medicine, University of Rome “Tor Vergata”, Via Montpellier 1, 00133 Rome, Italy; Neurology Unit, Department of Systems Medicine, University of Rome “Tor Vergata”, Via Montpellier 1, 00133 Rome, Italy; Neuroradiology Unit, Department of Biomedicine and Prevention, University of Rome “Tor Vergata”, Via Montpellier 1, 00133 Rome, Italy; Neurology Unit, Department of Systems Medicine, University of Rome “Tor Vergata”, Via Montpellier 1, 00133 Rome, Italy; UOSD Parkinson Centre, Tor Vergata University Hospital, Viale Oxford 81, 00133 Rome, Italy; Neurology Unit, Department of Systems Medicine, University of Rome “Tor Vergata”, Via Montpellier 1, 00133 Rome, Italy; Neurology Unit, Department of Systems Medicine, University of Rome “Tor Vergata”, Via Montpellier 1, 00133 Rome, Italy; Neurology Unit, Department of Systems Medicine, University of Rome “Tor Vergata”, Via Montpellier 1, 00133 Rome, Italy; UOSD Parkinson Centre, Tor Vergata University Hospital, Viale Oxford 81, 00133 Rome, Italy; Neurology Unit, Department of Systems Medicine, University of Rome “Tor Vergata”, Via Montpellier 1, 00133 Rome, Italy

**Keywords:** Parkinson’s disease, central autonomic network, functional connectivity, autonomic symptoms, EEG

## Abstract

Autonomic symptoms in Parkinson’s disease result from variable involvement of the central and peripheral systems, but many aspects remain unclear. The analysis of functional connectivity has shown promising results in assessing the pathophysiology of Parkinson’s disease. This study aims to investigate the association between autonomic symptoms and cortical functional connectivity in early Parkinson’s disease patients using high-density EEG. 53 early Parkinson’s disease patients (F/M 18/35) and 49 controls (F/M 20/29) were included. Autonomic symptoms were evaluated using the Scales for Outcomes in Parkinson’s disease–Autonomic Dysfunction score. Data were recorded with a 64-channel EEG system. We analyzed cortical functional connectivity, based on weighted phase-lag index, in θ-α-β-low-γ bands. A network-based statistic was used to perform linear regression between Scales for Outcomes in Parkinson’s disease–Autonomic Dysfunction score and functional connectivity in Parkinson’s disease patients. We observed a positive relation between the Scales for Outcomes in Parkinson’s disease–Autonomic Dysfunction score and α-functional connectivity (network τ = 2.8, *P* = 0.038). Regions with higher degrees were insula and limbic lobe. Moreover, we found positive correlations between the mean connectivity of this network and the gastrointestinal, cardiovascular, and thermoregulatory domains of Scales for Outcomes in Parkinson’s disease–Autonomic Dysfunction. Our results revealed abnormal functional connectivity in specific areas in Parkinson’s disease patients with greater autonomic symptoms. Insula and limbic areas play a significant role in the regulation of the autonomic system. Increased functional connectivity in these regions might represent the central compensatory mechanism of peripheral autonomic dysfunction in Parkinson’s disease.

## Introduction

Parkinson’s disease (PD) is a multi-system neurodegenerative disorder involving a widespread spectrum of motor and non-motor symptoms (NMS), with the latter often preceding the motor manifestations and significantly affecting the overall quality of life (Kalia and Lang 2015).

Autonomic symptoms (AS), including orthostatic hypotension (OH), constipation, delayed empty stomach, urinary, sexual dysfunction, and sweating problems, arise in PD due to a varying involvement of the autonomic nervous system (ANS).

Most PD patients experience at least 1 AS, with cardiovascular dysfunction, primarily manifesting as OH, postprandial hypotension but also encompassing supine hypertension, affecting up to 40% of PD patients (Jain and Goldstein 2012). However, the prevalence of AS at the early stage of PD is still debated. Indeed, previous studies showed that neurogenic OH (NOH) is not common at the early PD stage, and the prevalence of AS increases with age, disease duration, and use of drugs (Palma and Kaufmann 2020; Baschieri et al. 2021; Rocchi et al. 2021; Beach and McKay 2024). However, Goldestein et al. found that up to 60% of PD patients were affected by NOH before or within a year after the onset of motor symptoms (Goldstein 2006). Nevertheless, it is now recognized that an alteration of the ANS may occur at any stage of PD, including the premotor phase (Palma and Kaufmann 2014), and even in cases with minimal or no overt symptoms of dysautonomia (Palma and Kaufmann 2020). Recent neuropathological, imaging, and clinical evidence support the hypothesis that PD comprises 2 different subtypes, body-first and brain-first, based on the initial localization of the α-synuclein inclusions. In particular, in the body-first subtype, the aggregation would initiate in the enteric or peripheral ANS and then ascend through the vagus nerve to the brain (Horsager et al. 2020).

The ANS is a complex structure, including both central autonomic network (CAN) within the central nervous system (CNS) and peripheral components (Palma and Mano 2018). The autonomic dysfunction in PD is traditionally ascribed to a primary disruption of peripheral autonomic pathways. However, despite the fact that the central autonomic structures are also affected, their contribution to autonomic dysfunction remains controversial (Dayan et al. 2018).

A promising approach to improving the understanding of PD pathophysiology is the study of functional connectivity (FC) through different techniques (Conti, Bovenzi, et al. 2022). Among these, high-density EEG (HD-EEG), thanks to the use of novel approaches to analysis, allows a reliable network-based estimation of FC with high temporal resolution.

The purpose of this study was to explore the relationship between AS and cortical FC in a cohort of de novo PD patients using HD-EEG to evaluate the possible CNS involvement in PD-associated dysautonomia in the early stages of the disease.

## Materials and methods

### Study population

This study involved 50 patients (F/M 18/35) diagnosed with early-stage idiopathic PD and followed at the Parkinson Unit of the University of Rome “Tor Vergata” from June 2022 to March 2024. The inclusion criteria of the study were: (i) diagnosis of PD according to the 2015 MDS criteria (Postuma et al. 2015); (ii) a disease stage according to the Hoehn and Yahr (H&Y) scale ≤ 2 (Goetz et al. 2004); (iii) a disease duration < 48 mo; (iv) mild–moderate motor impairment as measured by a MDS Unified Parkinson’s Disease Rating Scale part 3 (MDS-UPDRS part III) ≤40; (v) limited dopaminergic medications, MAO-B inhibitors (MAOB-i) and/or low doses of dopamine agonists (pramipexole ≤ 0.52 mg, rotigonine ≤ 4 mg, ropirinole ≤ 4 mg); and (vi) no treatment with Levodopa (LD naïve), in order to overcome its possible long-term effects (Zappia and Nicoletti 2010).

Exclusion criteria were: (i) a history of epilepsy or other neurological conditions that might have caused an alteration of the EEG trace; (ii) a history of major psychiatric or other neurological diseases; (iii) dementia, defined as a Mini-Mental State Examination (MMSE) score <25 (Folstein et al. 1975); (iv) brain parenchymal lesions on brain MRI (e.g. brain tumors, strokes, abscesses, or other infectious conditions, etc.); and (v) the presence of systemic or metabolic diseases affecting the ANS, such as diabetes mellitus, heart diseases, renal failure, or the assumption of concurrent medications potentially affecting the autonomic function.

A group of 49 age- and sex-matched HCs (F/M 20/29) with no history of major neurological/psychiatric disorders and no other exclusion criteria were also included in the study.

Moreover, based on the value of the Scales for Outcomes in Parkinson’s disease–Autonomic Dysfunction (SCOPA-AUT) score, we divided the PD cohort into 2 subgroups: PD patients with mild AS (SCOPA-AUT ≤ 15, *n* = 30) and PD patients with moderate–severe AS (SCOPA-AUT > 16, *n* = 23).

Informed consent was obtained from the subjects involved in the study. The study was conducted in agreement with the principles of the Helsinki declarations. The ethical committee of Policlinico Tor Vergata approved the study (RS 17/18).

### Clinical assessments

Socio-demographic and clinical history data were collected from all patients and HCs on the same day as the EEG recording.

In PD patients, motor impairment was assessed using the H&Y scale (Goetz et al. 2004) and the MDS Unified Parkinson’s Disease Rating Scale Part 3 (MDS-UPDRS III) (Goetz et al. 2008). Furthermore, the motor subscores were calculated as the sum of the specific items of MDS-UPDRS-III (rigidity 3.3; bradykinesia 3.4 to 3.8, 3.14; tremor 3.15 to 3.18; gait/postural stability 3.9 to 3.13). The presence of rapid eye movement sleep behavior disorder (RBD) was investigated using the RBD screening questionnaire (RBD-SQ) (Stiasny-Kolster et al. 2007). Hyposmia was documented through an anamnestic investigation. Cognitive function was assessed through MMSE (Folstein et al. 1975) and Montreal cognitive assessment (MoCA) scales (Nasreddine et al. 2005). Finally, in each patient, ASs were investigated through the SCOPA-AUT scale (Visser et al. 2004). This scale is a specific 23-item self-completed questionnaire for the assessment of autonomic dysfunction in patients with PD, divided into 6 main domains: gastrointestinal, urinary, cardiovascular, thermoregulatory, pupillomotor, and sexual. All clinical evaluations were performed after an overnight withdrawal from any dopaminergic drug. The levodopa equivalent daily dose (LEDD, mg/d) was calculated using a conventional formula for all PD patients. This study was conducted in accordance with the principles of the Helsinki Declaration. The local ethics committee approved the study. Informed consent was obtained from each participant in this study.

### E‌EG recording

HD-EEG data were recorded for 10 min at a sampling rate of 1024 Hz, band-pass filtered at 0.5 to 50 Hz using a 64-channel EEG system (EbNeuro BePlus-ProStandard). Electrodes were positioned according to the 10-10 International System (Nuwer 2018). The impedance was kept below 5 kΩ. HD-EEG recording was performed in the eyes-closed (EC) resting state: the subjects were instructed to keep their eyes closed while staying awake for 5 min (Yassine et al. 2022). Then, reactivity to eye opening and activation tests were performed to exclude the appearance of epileptiform elements in the EEG data.

In the PD group, the HD-EEG recording was performed after overnight withdrawal from the last administration of dopaminergic therapy. The same HD-EEG recording was obtained in HC.

### FC analysis

HD-EEG recordings were segmented into epochs of 30 s each for visualization purposes (Yassine et al. 2022). The first epoch of each recording was discarded. Then, we manually selected the first 6 consecutive low-artifact epochs (total 180 s) that were retained for the following analysis. The same method was used for PD patients and HCs.

Independent component analysis (ICA) was used to remove the residual EEG artifacts (Hyvärinen and Oja 2000). Then, we proceeded with EEG source localization (Conti, Stefani, et al. 2022). To this purpose, all subjects underwent MRI, and personal T1-weighted MPRAGE MRI sequence and EEG data were co-registered through the identification of the same anatomical landmarks, and the Boundary Element Method (BEM) (Jatoi et al. 2016) was used to solve the forward problem. We used weighted minimum-norm estimation (wMNE) to solve the inverse problem (Grech et al. 2008). The sources obtained were divided into 68 brain regions using the Desikan–Killiany atlas (Desikan et al. 2006).

FC was calculated in source space using weighted phase lag index (wPLI), a measure known to reduce conduction volume artifacts, noise artifacts, and bias from small samples (Hardmeier et al. 2014). Phase information from the preprocessed signals was computed using the Hilbert transform in θ (4 to 8 Hz), α (8 to 13 Hz), β (13 to 30 Hz), and low-γ (30 to 45 Hz) frequency bands. Dynamic FC matrices were computed between any pair of regions in the different EEG bands on segments of 1-s length, with an overlap of 50%, according to Welch’s method. Of note, this method is not affected by the length of the displayed epochs (Jwo et al. 2021). Those matrices were averaged across time epochs to obtain static FC matrices.

Analysis was made using Brainstorm toolbox (Tadel et al. 2011), combined with custom-written scripts for MATLAB R2023b. Other details of the methodology we used can be found in this paper by our group (Conti et al. 2023).

### Statistical analysis

Differences in qualitative and quantitative variables between PD patients and HCs and between PD patients with mild and moderate–severe AS were tested using the Chi-square test and two-sample *t*-test, respectively.

Differences in θ, α, β, and low-γ FC between PD patients and HCs were analyzed using the network-based statistic (NBS). NBS is a cluster-based statistical method used in numerous previous studies (De Micco et al. 2021; Yassine et al. 2022) that has been shown to provide greater statistical power than standard univariate tests and traditional correction methods (Zalesky et al. 2010). Furthermore, NBS, as a generalized linear method (GLM), was also used to study linear regression between FC and SCOPA-AUT total scores of PD patients. In our experimental design, we used 2.0 as the initial threshold *t* and 5,000 permutations for the permutation test. Then, for each NBS analysis, we selected the network with the lowest *t* value and *P* < 0.05 in order to identify the largest differential network with statistical significance. Age, sex, disease duration, and MDS-UPDRS III were considered confounding factors in NBS.

Moreover, we defined the mean network connectivity (mNC) of the disrupted network as the average link connectivity of networks from the NBS analysis. A two-sample *t*-test was used to evaluate differences in mNC of the disrupted network between PD patients with mild and moderate–severe AS. Finally, the mNC of the network related to the SCOPA-AUT total score was correlated using Pearson correlation with the SCOPA-AUT total score and each item of the SCOPA-AUT.

No statistical power calculation was conducted prior to the study. The sample size was based on the available data and on our previous experience with the design of an FC study based on NBS. The statistical analysis was performed using MATLAB 2023b, NBS (Zalesky et al. 2010) toolboxes. Graphs were based on custom-written scripts in MATLAB 2023b and R (ggplot2 package).

## Results

The demographic and clinical characteristics of PD patients and HCs are reported in Table 1. No significant differences emerged between PD patients and HCs. The demographic and clinical features of PD patients with mild (SCOPA-AUT ≤ 15) and moderate–severe (SCOPA-AUT > 16) AS are shown in Table 2*.* PD patients with moderate–severe AS are characterized by significantly higher MDS-UPDSR III total score (*P* = 0.032), bradykinesia subscore (*P* = 0.017), gait subscore (*P* = 0.003), and LEDD (*P* = 0.033) compared to PD patients with mild AS. Moreover, RBD was significantly more frequent in PD patients with moderate–severe AS compared to those with mild AS (*P* = 0.015).

**Table 1:** Clinical and demographic data of the study population.

	**PD patients** **(*n* = 50)**	**HCs** **(*n* = 49)**	***P*-value**
**Age (y)**	60.7 ± 10.7	59.4 ± 12.3	0.58
**Sex (F/M)**	17/33	20/29	0.48
**Disease duration (mo)**	22.5 ± 15.5	—	—
**BMI**	25.7 ± 3.8	24.2 ± 4.6	0.08
**Laterality (L/R)**	24/26	—	—
**RBD (Y/N)**	18/32	—	—
**Hyposmia (Y/N)**	20/30	—	—
**H&Y stage**	1.8 ± 0.4	—	—
**MDS-UPDRS-III**	26.0 ± 9.6	—	—
Rigidity	5.4 ± 3.0	—	—
Bradykinesia	12.0 ± 6.3	—	—
Gait/postural	2.6 ± 1.9	—	—
Tremor	4.0 ± 3.6	—	—
**Total LEDD (mg/die)**	101.2 ± 99.9	—	—
**MoCA**	26.4 ± 2.9	—	—
**MMSE**	28.0 ± 2.4	—	—
**SCOPA-AUT**	15.7 ± 10.9	—	—
Gastrointestinal	3.6 ± 3.6	—	—
Urinary	6.4 ± 4.4	—	—
Cardiovascular	0.9 ± 1.4	—	—
Pupillomotor	1.0 ± 1.6	—	—
Thermoregulatory	1.7 ± 2.5	—	—
Sexual	2.2 ± 2.9	—	—

Data are expressed as mean ± standard deviation of variables. Age is expressed in years. Disease duration is expressed in months.

**Table 2:** Clinical and demographic data of PD patients with mild (SCOPA-AUT ≤ 15) and moderate–severe (SCOPA-AUT > 16) AS.

	**PD patients with** **SCOPA-AUT ≤ 15** **(*n* = 28)**	**PD patients with** **SCOPA-AUT > 16** **(*n* = 22)**	***P*-value**
**Age (y)**	59.4 ± 10.0	62.5 ± 11.6	0.32
**Sex (F/M)**	10/18	7/15	0.77
**Disease duration (mo)**	19.8 ± 14.4	25.8 ± 16.4	0.17
**BMI**	25.4 ± 4.3	26.1 ± 3.2	0.57
**Laterality (L/R)**	12/16	14/8	0.14
**RBD (Y/N)**	6/22	10/12	0.015^*^
**Hyposmia (Y/N)**	10/18	10/12	**0.49**
**H&Y stage**	1.8 ± 0.4	1.9 ± 0.3	0.42
**MDS-UPDRS-III**	23.5 ± 9.4	26.1 ± 3.2	0.032^*^
Rigidity	4.9 ± 3.0	6.1 ± 3.1	0.17
Bradykinesia	10.1 ± 5.9	14.4 ± 6.3	0.017^*^
Gait/postural	1.9 ± 1.6	3.5 ± 1.9	0.003^*^
Tremor	4.3 ± 3.5	3.7 ± 3.7	0.59
**Total LEDD (mg/die)**	74.7 ± 92.5	135.0 ± 100.7	0.033^*^
**MoCA**	26.9 ± 2.3	25.9 ± 3.5	0.25
**MMSE**	28.2 ± 1.8	27.7 ± 3.1	0.48

Data are expressed as mean ± standard deviation of variables. Age is expressed in years. Disease duration is expressed in months. Significant differences were marked in bold and with ^*^.

### Altered FC networks in PD patients compared to HCs

Through the NBS analysis, we identified a statistically significant network at θ band (*t* = 2.3, *P* = 0.031), where the FC was lower in PD than HCs. The network comprised 49 nodes and 74 links and was rather lateralized in the right hemisphere (54.7%). ROIs with higher degrees were part of the right orbitofrontal cortex, right insula, ventrolateral prefrontal cortex (left pars triangularis and right pars orbitalis), sensorimotor (bilateral postcentral and right precentral cortex), and limbic (mainly right anterior cingulate area) lobes*.* Moreover, mNC was significantly lower in PD compared to HC (*t* = 5.0, *P* < 0.001).

In addition, we found a statically significant network at α band (*t* = 2.3, *P* = 0.033), where the connectivity was lower in PD than HCs. This network was composed of 52 nodes and 136 links and was slightly lateralized in the left hemisphere (51.8%). ROIs with higher degrees were part of dorsolateral, ventrolateral and dorsoventral prefrontal cortex (bilateral superior frontal, left caudal middle frontal, and right pars opercularis ROIs), orbitofrontal cortex (bilateral medial and lateral orbitofrontal areas), sensorimotor (mostly left paracentral and right postcentral), temporal (predominantly left superior temporal), and limbic (mainly bilateral anterior cingulate) lobes. Furthermore, mNC was significantly lower in PD compared to HC (*t* = 4.7, *P* < 0.001).

On the contrary, a significant network was found in β band (*t* = 2.5, *P* = 0.039), characterized by hyperconnectivity in PD patients compared to HCs. The network comprised 53 nodes and 85 connections and was slightly predominant in the left hemisphere (52.6%). Brain regions with higher degrees were part of the sensorimotor (mainly bilateral precentral and postcentral), limbic (predominantly bilateral anterior cingular cortex), and parietal (mostly bilateral precuneus) lobes. Likewise, mNC was significantly higher in PD than HC (*t* = −4.8, *P* < 0.001).

No differences were found in the low-γ band between PD patients and HCs.

### Relations between FC and SCOPA-AUT score in PD patients

**Fig. 1:**
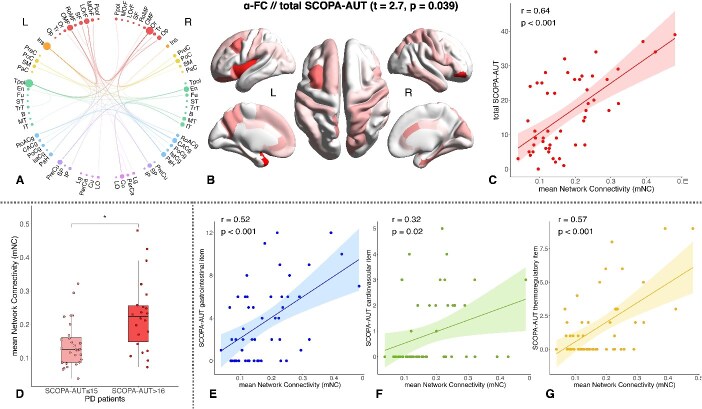
A) Graph representations of the network positively related to the SCOPA-AUT total score in PD patients in α band. Colors indicate different brain lobes: Red = frontal, orange = insula, yellow = sensorimotor, green = temporal, cyan = limbic, purple = parietal, magenta = occipital; circle diameters are directly proportional to region of interest (ROI) degrees. B) Representation of ROI degrees on model brain surface of the α-FC//total SCOPA-AUT network. D) Difference of mNC between PD patients with mild (SCOPA-AUT ≤ 15) and with moderate–severe AS (SCOPA-AUT > 16). Correlations between the mNC of the α-FC//total SCOPA-AUT network and C) the SCOPA-AUT total score, E) the gastrointestinal item, F) the cardiovascular item, and G) the thermoregulatory item.

We performed NBS-based linear regression to study the relationship between FC at each band and AS of our early PD population, assessed by the SCOPA-AUT score. We found a positive regression between FC at α band and SCOPA-AUT total score (*t* = 2.7, *P* = 0.039). This network comprised 42 nodes and 67 links and was slightly predominant in the right hemisphere (53.5%). ROIs with higher degrees were part of the insular cortex (in particular left insula), limbic (mainly bilateral anterior cingulate and right posterior cingulate), parietal (in particular left precuneus), and prefrontal (mostly left dorsolateral and right orbitofrontal cortex) lobes (Fig. 1A and B). Moreover, mNC was positively correlated with the SCOPA-AUT total score (*r* = 0.64, *P* < 0.001) (Fig. 1C)*.* Finally, we found that mNC of this network was significantly higher in PD patients with moderate–severe AS compared to those with mild AS (*T* = 3.31, *P* = 0.002) (Fig. 1D)*.*

No significant relations were observed between SCOPA-AUT and EEG-FC in the other bands.

Finally, we examined the Pearson correlations between the mNC of this dysautonomic-related FC network and each item of the SCOPA-AUT score, aiming to identify the specific items most strongly associated with the dysfunction. We found significant positive correlations between mNC and the gastrointestinal (*r* = 0.52, *P* < 0.001) (Fig. 1D), the cardiovascular (*r* = 0.32, *P* = 0.020) (Fig. 1E), and the thermoregulatory (*r* = 0.57, *P* < 0.001) (Fig. 1F) items. Conversely, no significant correlations were observed with the urinary, pupillomotor, and sexual items.

## Discussion

In this study, we analyzed the functional features of early-stage PD patients using HD-EEG, investigating the changes in PD cortical connectivity with respect to the AS burden. Our study arose from recent literature exploring altered cortical connectivity in PD de novo and its association with motor and NMS (Conti et al. 2023) and its reliability as a prognostic value (De Micco et al. 2024). Here, we highlighted the possible relation with AS as a novelty element.

First, we observed how PD patients, compared to HCs, were characterized by a reduced FC in the θ band, mainly in prefrontal, insular, and sensorimotor areas, a reduced FC in the α band, mostly in prefrontal, sensorimotor, temporal, and limbic regions, and an increased FC in the β band, predominantly in the sensorimotor, limbic, and parietal areas. These findings confirmed the previous results on a larger sample, showing how early-stage PD patients may present with band- and network-specific functional changes within several cortical areas (Conti et al. 2023).

Nevertheless, the core finding of the present study was the positive correlation between the total SCOPA-AUT score and FC changes in the α band in several brain regions of early-stage PD patients. Notably, through the NBS analysis, we identified a dysautonomic-related network, mostly involving the insular and cingulate cortex, that resulted in being hyperactivated in relation to dysautonomic symptoms.

Previous studies based on fMRI have identified an intricate system of brain regions, constituting the CAN, that plays a crucial role in integrating afferent signals and controlling the function of the ANS (Sklerov et al. 2019). Several cortical and subcortical structures have been included in CAN, such as the insula, cingulate cortex, hippocampus, cerebellum, thalamus, and, other than the brainstem. Consistently, the dysautonomic-related network we found in the α band showed a significant overlap with these areas, further enlightening their role in ANS control since the earliest stages of PD.

Here, we found that the functional alteration linked to AS appeared to be specific to the α band. The hypoconnectivity in the α band, mainly in prefrontal and sensorimotor regions, seems to be a characteristic feature in PD patients compared to HCs, as reported in previous studies and further corroborated in this research. Moreover, α-FC disruption in PD has been linked to cognitive impairment (Babiloni et al. 2018) and gait disturbances (Conti et al. 2023). Nevertheless, here we found that the overall autonomic burden was associated with increased connectivity rather than a disruption in the α band, even if limited to a few specific areas, such as the insula and cingulate cortex. These results represent novel findings that deserve further discussion.

According to the recent “brain-first versus body-first model” of PD, the α-synuclein pathology starts in a single location, the body or the brain, and then propagates symmetrically or asymmetrically, depending on the origin of the accumulation, defining “brain first” and “body first” PD (Horsager et al. 2020). Thus, the subgroup of patients known as “brain first” is supposed to have an initial α-synuclein pathology that arises inside the CNS. Instead, “body-first” PD patients, where synucleopathy originates peripherally, are those with the greater and early burden of constipation, REM behavior disorders (RBD), and AS. Consistently with the “brain versus body first” theory, we could hypothesize that PD patients with an initially higher burden of AS, probably mostly framed in a body first subtype, could have a pathway characterized by hyperconnectivity limited to the CAN as a central compensatory mechanism of peripheral autonomic dysfunction, almost at the early stage of the disease. In line with this hypothesis, we found that the group of PD patients with moderate–severe AS (SCOPA-AUT > 16) had a significantly higher prevalence of RBD compared to PD patients with mild AS (SCOPA-AUT ≤ 15). Indeed, RBD in the early stages of the disease is emerging as a robust marker of the body-first subtype (Horsager et al. 2019). Otherwise, it cannot be excluded that the α hyperconnection we found in CAN of PD patients with a higher AS burden could reflect central co-pathology as a disarrangement of central autonomic control that can be seen since the early stages of PD with AS.

However, the α band specificity of such CAN alteration is challenging to discuss, even considering that until today, very few studies, mostly based on resting-state MR functional imaging (rsfMRI), have investigated FC changes associated with dysautonomia in PD (Dayan et al. 2018; Tessa et al. 2019), while no studies have based on EEG connectivity. HD-EEG is an innovative tool that can directly assess cortical activity with a high temporal resolution, as opposed to fMRI, which is based on indirect measures of neural activity with a suboptimal temporal resolution to capture brain dynamics, which occur in a few milliseconds. On the contrary, EEG has the significant disadvantage of a less spatial resolution compared to fMRI.

We thus analyzed the correlations between the mNC of the dysautonomic-related network and each subitem of the SCOPA-AUT scale, and we observed that the more significant associations with the abnormal central FC were found for the thermoregulatory, gastrointestinal, and cardiovascular items, while there was no correlation with urinary, pupillary, and sexual-function items.

These results align with our previous hypothesis of a central compensatory mechanism for peripheral autonomic dysfunction. Indeed, all AS investigated with the SCOPA-AUT may appear at the early stage of disease, but symptoms of dysfunctional thermoregulatory, gastrointestinal, and cardiovascular systems are known to underlie a primary involvement of peripheral structures. Actually, 123I-MIBG imaging shows impaired postganglionic noradrenergic fibers in PD patients with abnormal cardiovascular function (Katagiri et al. 2015). Also, vagus nerve degeneration, together with enteric neuropathology and microbiota imbalance, has been observed in PD patients with gastrointestinal impairment (Xu et al. 2022; Schirinzi et al. 2023). Otherwise, the pathophysiology of sudomotor dysfunction is still being debated since previous studies have demonstrated both a central and peripheral origin of the disorder in the early stages of PD (Hirashima et al. 1996; Rocchi et al. 2021). However, the peripheral intraepidermal nerve fiber degeneration, in parallel with skin α-synuclein accumulation, would appear to contribute to the sudomotor and vasoconstrictive dysfunctions (Chen et al. 2020). Still, the insula, amygdala, anterior cingulate cortex, and the brainstem’s reticular formation exert control of these functions throughout descending pathways (Asahina et al. 2014), consistently with our hypothesis of the central compensation of this area to peripherally altered structures. On the contrary, urinary and sexual dysfunction seem mainly caused by central mechanisms in PD (Chen et al. 2020), especially dopaminergic imbalance. It is known that the frontal-basal ganglia dopaminergic alteration results in the disinhibition of the micturition reflex and subsequent detrusor overactivity and overactive bladder symptoms (Sakakibara et al. 2016; Kim and Jun 2019).

This study has some limitations. The main limitation of this study is the lack of objectionable measures of dysautonomia, since we evaluated AS through a subjective, self-reported scale. However, the SCOPA-AUT score has been shown to be a reliable and valid questionnaire to assess autonomic dysfunction in PD (Visser et al. 2004). Furthermore, several other studies have used it to measure AS (Chaudhuri and Schapira 2009; Pavelka et al. 2022), and it was also correlated with fMRI in PD (Dayan et al. 2018). Moreover, our PD population was composed of LD-naive de novo PD patients, or PD patients at most under treatment with MAOIs or low doses of dopamine agonists. Hence, this recruitment approach minimizes the effect of dopaminergic drugs on EEG-FC, but it cannot be completely removed. Furthermore, the inclusion of patients with relatively high MDS-UPDRS Part III scores might represent a bias, supposing that the burden of motor impairment in de novo PD patients could overlap with AS. However, we excluded patients with higher motor symptoms (MDS-UPDRS-III > 40) and also considered this score as a covariate. Finally, the low-artifact epochs were manually selected, introducing potential bias. However, most of the computation was done using automatic functions integrated in high-level functions in MATLAB without operator intervention.

## Conclusion

This study found differences in brain FC in early-stage PD according to the extension of the AS burden. It opens a window into the role of central control of the ANS by non-invasive, low-cost detection of FC in PD patients. It is conceivable that these findings could help better understand the pathophysiology of AS in PD. Moreover, discovering specific brain regions functionally linked to dysautonomia in PD could be useful in enveloping novel therapeutic protocols based on repetitive transcranial magnetic stimulation (rTMS) or transcranial electrical stimulation (TES) to treat AS. Further studies, integrated with objective autonomic assessment tools, are needed to explore the issue of FC of CAN in patients with PD at different stages of the disease.

## Data Availability

The raw data supporting the conclusions of this article will be made available by the authors, upon reasonable request.
